# Predicting achievement of clinical goals using machine learning in myasthenia gravis

**DOI:** 10.1371/journal.pone.0330044

**Published:** 2025-08-14

**Authors:** Hiroyuki Akamine, Akiyuki Uzawa, Satoshi Kuwabara, Shigeaki Suzuki, Yosuke Onishi, Manato Yasuda, Yukiko Ozawa, Naoki Kawaguchi, Tomoya Kubota, Masanori P. Takahashi, Yasushi Suzuki, Genya Watanabe, Takashi Kimura, Takamichi Sugimoto, Makoto Samukawa, Naoya Minami, Masayuki Masuda, Shingo Konno, Yuriko Nagane, Kimiaki Utsugisawa

**Affiliations:** 1 Department of Neurology, Graduate School of Medicine, Chiba University, Chiba, Japan; 2 Department of Neurology, Keio University School of Medicine, Tokyo, Japan; 3 Department of Neurology, Neurology Chiba Clinic, Chiba, Japan; 4 Division of Health Sciences, Department of Clinical Laboratory and Biomedical Sciences, The University of Osaka Graduate School of Medicine, Suita, Osaka, Japan; 5 Department of Neurology, National Hospital Organization Sendai Medical Center, Sendai, Japan; 6 Department of Neurology, Hyogo Medical University, Nishinomiya, Japan; 7 Department of Clinical Neuroscience and Therapeutics, Hiroshima University, Hiroshima, Japan; 8 Department of Neurology, Kindai University Faculty of Medicine, Osakasayama, Japan; 9 Department of Neurology, National Hospital Organization Hokkaido Medical Center, Sapporo, Japan; 10 Department of Neurology, Tokyo Medical University, Tokyo, Japan; 11 Department of Neurology, Toho University Ohashi Medical Center, Tokyo, Japan; 12 Department of Neurology, Hanamaki General Hospital, Hanamaki, Japan; Frederick National Laboratory for Cancer Research, UNITED STATES OF AMERICA

## Abstract

**Background:**

Myasthenia Gravis (MG) is an autoimmune disease characterized by the production of autoantibodies against neuromuscular junctions, leading to varying degrees of severity and outcomes among patients. This variability makes clinical evaluation crucial for determining appropriate treatment targets. However, accurately assessing Minimal Manifestation (MM) status is challenging, requiring expertise in MG management. Therefore, this study aims to develop a diagnostic model for MM in MG patients by leveraging their clinical scores and machine learning approaches.

**Methods:**

This study included 1,603 MG patients enrolled from the Japan MG Registry in the 2021 survey. We employed non-negative matrix factorization to decompose three MG clinical scores (MG composite score, MGADL scale, and MG quality of life (QOL) 15r) into four distinct modules: Diplopia, Ptosis, Systemic symptoms, and QOL. We developed a machine learning model with the four modules to predict MM or better status in MG patients. Using 414 registrants from the Japan MG Registry in the 2015 survey, we validated the model’s performance using various metrics, including area under the receiver operating characteristic curve (AUROC), sensitivity, specificity, accuracy, F1 score, and Matthews Correlation Coefficient (MCC).

**Results:**

The ensemble model achieved an AUROC of 0.94 (95% CI: 0.94–0.94), accuracy of 0.87 (95% CI: 0.86–0.88), sensitivity of 0.85 (95% CI: 0.85–0.86), specificity of 0.89 (95% CI: 0.88–0.91), precision of 0.93 (95% CI: 0.92–0.94), F1 score of 0.89 (95% CI: 0.88–0.89), and MCC of 0.74 (95% CI: 0.72–0.75) on the validation dataset.

**Conclusions:**

The developed MM diagnostic model can effectively predict MM or better status in MG patients, potentially guiding clinicians in determining treatment objectives and evaluating treatment outcomes.

## Introduction

Myasthenia Gravis (MG) is an autoimmune disease that produces autoantibodies targeting acetylcholine receptors (AChR) at neuromuscular junctions [1]. Approximately 80% of patients have antibodies against AChR, while 5–8% produce muscle-specific kinase (MuSK) antibodies [2]. Around 10% of MG cases are called double seronegative MG, as they do not have detectable levels of either pathogenic antibodies [3]. MG is a disease characterized by muscle weakness affecting various parts of the body, leading to symptoms such as diplopia, ptosis, dysarthria, dysphagia, respiratory distress, limb weakness, and fatigue [1]. A notable feature of this disease is the fluctuation of these symptoms within a day or across different days. Several standardized scales are widely used to evaluate the clinical symptoms of MG patients: the quantitative MG (QMG) scale [4] for objective assessment of muscle weakness severity, the MG activities of daily living (MGADL) score [5] for evaluating functional limitations in daily activities, and the MG composite (MGC) scale [6] as a comprehensive measure combining both objective and subjective assessments. MG patients often experience not only motor symptoms but also difficulties in psychological and social well-being [7]. However, it is challenging to assess these burdens. MG Quality of Life 15 (MGQOL15) [8] and its revised version (MGQOL15r) [9] are able to capture a more holistic, patient-centered assessment [10]. The MG Foundation of America (MGFA) classification is widely used to evaluate treatment outcomes by categorizing pre-treatment conditions into five stages and post-treatment status into eight stages [11]. In clinical practice, many physicians set Minimal Manifestation (MM) status as a treatment goal based on international guidelines [12]. Jaretzki III et al. defined “MM status: The patient has no symptoms or functional limitations from MG but has some weakness on examination of some muscles” [11]. This ambiguity makes it challenging to establish clear cutoff values for defining MM in MG clinical scores. There is no clear definition to identify minimal muscle weakness that is clinically important, which can result in variability in how physicians interpret and apply the MM criteria. This inconsistency may lead to different treatment approaches and creates challenges in comparing outcomes across medical institutes. By providing visualization of MG symptoms in MM determination and standardizing the assessment process, our approach aims to support more unified treatment selection criteria and help identify unmet clinical needs in MG management. We aimed to develop a diagnostic model for MM using MG clinical scores from patients enrolled in the MG registry, with the goal of standardizing MM assessment.

## Methods

### Data collection and access

The Japan MG Registry 2021 survey [13] was conducted between April and October 2021 across 13 medical institutions, which collaboratively gathered clinical data from MG patients. Written informed consent was obtained from all participants. Each participant was assigned a study-specific identification number by one (Y.N.) of co-authors, and personally identifiable information was anonymized. The first author (H.A.) accessed the dataset on December 10, 2021. The clinical assessments were performed by neurologists with extensive experience in MG diagnosis and management.

### Data preprocessing

**Fig 1:**
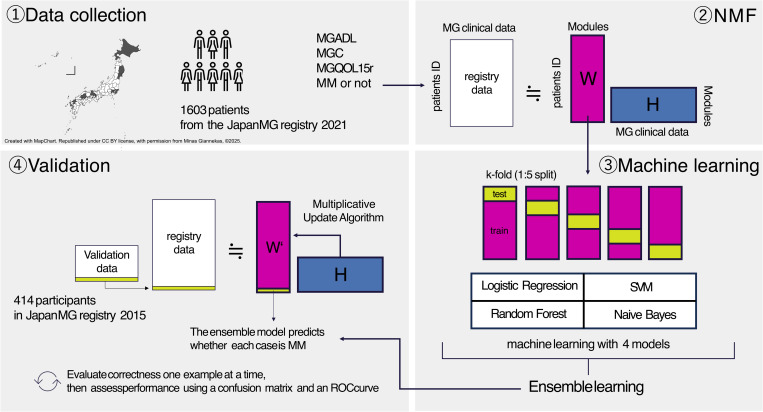
Overview of research methods. The outline of the research was visualized by classifying it into four categories: data collection, NMF, machine learning, and validation.

A total of 1,603 MG patients were included in the analysis after excluding cases with missing data from the 1,710 MG patients enrolled in the Japan MG Registry in the 2021 survey [13]. Their clinical scores, including MGC scale, MGADL score, and MGQOL15r, were collected. The evaluation of these clinical scores, as well as the determination of whether a patient had reached MM or better status (MM, Complete Stable Remission, or Pharmacologic Remission), was conducted by neurologists with expertise in MG management. To ensure consistency in feature scaling across the dataset, numerical columns were normalized to a range between 0 and 1. We utilized clinical data from 414 patients in the 2015 survey as the validation dataset. This ensured temporal independence between the training and validation datasets, allowing for a more robust evaluation of model. Fig 1 shows an overall view of Methods.

### Non-Negative Matrix Factorization (NMF)

Following the initial data preprocessing, we applied NMF [14] to decompose the dataset into latent modules. NMF was selected due to its ability to produce interpretable, non-negative components that align with the underlying structure of the data. We initialized the NMF model with four components using the nndsvd initialization method and a maximum of 200 iterations to ensure convergence. The decomposition resulted in two matrices: the W matrix (representing the latent variables for each sample) and the H matrix (representing the loadings of each feature on these latent variables). Both matrices were saved for downstream analysis and clustering.

### Hierarchical clustering and visualization

To further analyze the relationships between clinical features and their respective NMF-derived components, hierarchical clustering was performed on both the rows and columns of the H matrix. Using complete linkage and Euclidean distance as the metric, we reordered the H matrix based on the hierarchical clustering results. This reordered matrix was then visualized as a heatmap, which allowed us to visually inspect the clustering patterns and relationships between the clinical features and the four NMF modules.

### Model training and cross-validation

The W matrix obtained from the NMF decomposition was used to train machine learning models for predicting patient outcomes, the binary classification of achieving MM or better status. The dataset was split using 5-fold cross-validation, implemented via the K Fold method from scikit-learn. For each fold, the training and test sets were extracted, and four machine learning models were trained: Support Vector Machine (SVM), Logistic Regression, Random Forest, and Naive Bayes. The models were optimized using GridSearchCV for SVM, Logistic Regression, and Random Forest, while Naive Bayes was trained directly without parameter tuning. Specifically, for SVM, parameters such as ‘C’, ‘kernel’, and ‘gamma’ were tuned; for Logistic Regression, different configurations of ‘penalty’, ‘C’, ‘fit_intercept’, and ‘solver’ were explored, including support for elastic net regularization; and for Random Forest, ‘n_estimators’, ‘max_depth’, ‘min_samples_split’, and ‘min_samples_leaf’ were optimized. For each dataset split, the best parameters and scores were recorded. The final performance was assessed based on the average Area Under the Receiver Operating Characteristic Curve (AUROC) across five iterations. 95% confidence intervals were calculated with each model from 5-fold cross-validation.

### Model ensembling

To improve prediction accuracy, we used an ensemble learning approach with four models: SVM, Logistic Regression, Random Forest, and Naive Bayes. For performance evaluation including AUROC, we applied a soft voting strategy, averaging the predicted probabilities from all four models to generate the final prediction scores.

### Validation on independent dataset

To evaluate the generalization capability of the trained models, an independent dataset from 414 registrants from the Japan MG Registry in the 2015 survey was introduced for validation. The baseline characteristics of the validation cohort are presented in S1 Table. The validation dataset was preprocessed similarly to the training data, including feature scaling and variable selection. Using the H matrix obtained from NMF on the training data, we calculated the W matrix for the validation set with multiplicative updates, preserving the latent structure. The trained models were then applied to the validation data, and predictions were made using a majority voting ensemble approach. We evaluated the model’s performance using various metrics, including AUROC, sensitivity, specificity, accuracy, F1 score, and Matthews Correlation Coefficient (MCC).

### Ethical publication statement

We confirm that we have read the Journal’s position on issues involved in ethical publication and affirm that this report is consistent with those guidelines.

### Ethics approval

This study was approved by the ethics committee of each neurological center (Institutional Review Board No. R3-1 at Hanamaki General Hospital, the primary investigating institute) and written informed consent was obtained from all study participants.

## Results

### Data profiles

The profiles of the 1,603 MG patients included in this study are shown in Table 1. A total of 942 patients from the 1,603 MG patients achieved MM or better. The patients who achieved MM or better were more often male (46% vs. 30%), had a higher age of onset (51 [36, 64] vs. 46 [33, 60] years), and tended to be ocular type (29% vs. 11%). Whereas patients with SNMG and MuSK MG were less likely to achieve MM or better. Median MG clinical scores [IQR] were 5 [4, 8] vs. 1 [0, 2] for MGADL, 7 [4, 11] vs. 1 [0, 3] for MGC, and 12 [7, 18] vs. 3 [1, 7] for MGQOL15r in the non MM group and the MM or better group, significantly lower scores in the MM group (p < 0.001). MGFA PIS was 7.5%, PR 11%, and MM 81% in the MM or better group. The median PSL dose [IQR] was 5 mg [0, 8] in the non-MM group and 2 mg [0, 5] in the MM group, significantly lower in the MM group (p < 0.001).

**Table 1:** The profiles of 1,603 MG patients included in the study.

	Non MM	MM or better	p-value
	N = 661	N = 942	
Sex (Male)	197 (30%)	436 (46%)	< 0.001
Age	58 (48, 72)	62 (49, 73)	0.014
Onset age	46 (33, 60)	51 (36, 64)	< 0.001
MG subtypes			< 0.001
EOMG	148 (22%)	195 (21%)	
LOMG	135 (20%)	190 (20%)	
MuSK	30 (4.5%)	18 (1.9%)	
OMG	70 (11%)	275 (29%)	
SNMG	127 (19%)	56 (5.9%)	
TAMG	151 (23%)	208 (22%)	
MGFA PIS			< 0.001
CSR	0 (0%)	71 (7.5%)	
PR	0 (0%)	106 (11%)	
MM	0 (0%)	765 (81%)	
E	9 (1.4%)	0 (0%)	
I	485 (73%)	0 (0%)	
U	138 (21%)	0 (0%)	
W	27 (4.1%)	0 (0%)	
MGC total score	7 (4, 11)	1 (0, 3)	< 0.001
MGADL total score	5 (4, 8)	1 (0, 2)	< 0.001
MGQOL15r total score	12 (7, 18)	3 (1, 7)	< 0.001
PSL dose	5.0 (0.0, 8.0)	2.0 (0.0, 5.0)	< 0.001

The patients with MGFA PIS of CSR, PR, or MM at the time of the survey were defined as MM or better group; Median (IQR), n (%) is shown and statistical tests were performed with Wilcoxon rank sum test and Pearson’s Chi-squared test. minimal symptoms; MM, MG Foundation of America; MGFA, MG activities of daily living; MGADL, MG composite; MGC, MG quality of life 15 revised; MGQOL15r.

### NMF decomposition and feature clustering

**Fig 2:**
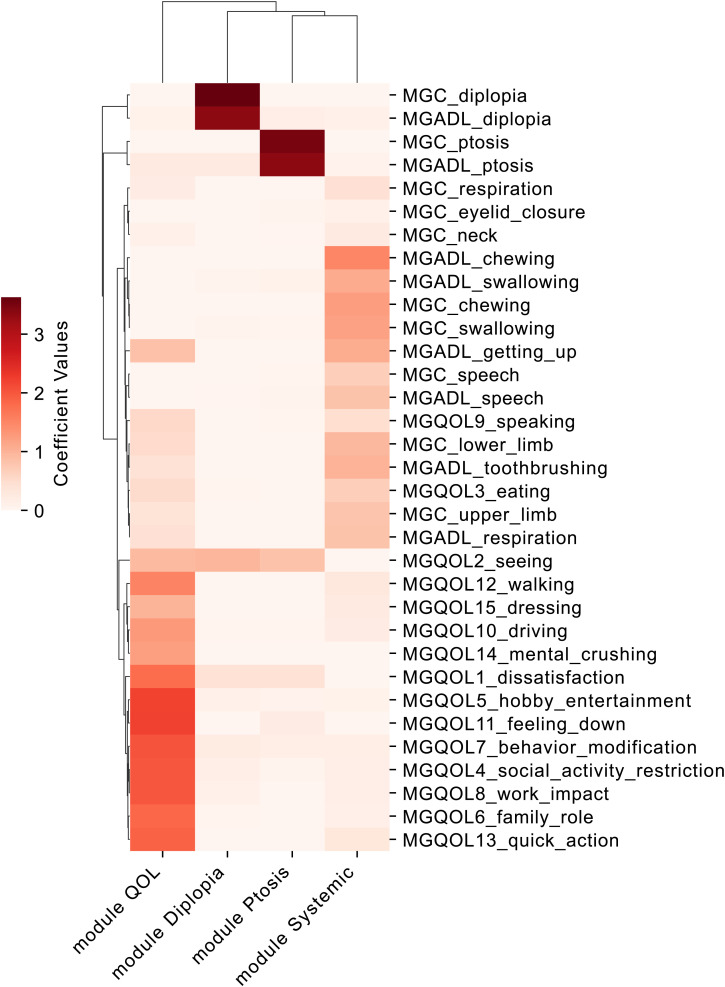
The H matrix obtained from NMF decomposition. The H matrix represents the four modules and their coefficients for each clinical score. Clinical parameters were clustered into modules defined as QOL, systemic symptoms, diplopia, and ptosis. The color of each cell indicates the coefficient value: cells with a red color have higher coefficient values, indicating that the clinical score is more strongly associated with that module.

**Fig 3:**
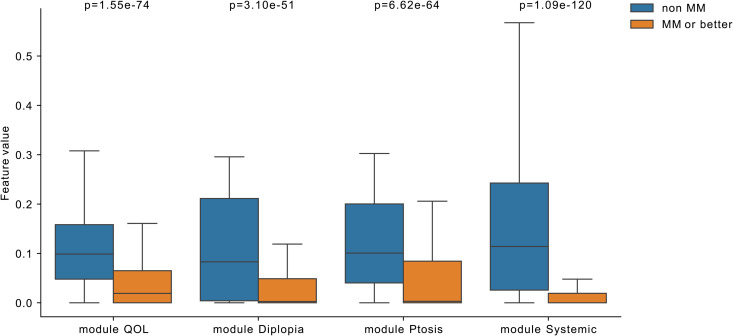
The distribution of each module in the non-MM and MM or better groups. The four modules of the two groups are compared in the boxplot. Each box represents the distribution of a feature for a group. The central line within each box indicates the median value, and the box’s length represents the interquartile range, giving an idea of the data’s spread. The statistical significance of the differences in distribution for each feature between the two groups were tested using the Mann−Whitney U test.

NMF was applied to the dataset of 1,603 patients, resulting in the decomposition of clinical features into four modules. The W matrix, which represents patient scores for latent variables, and the H matrix, which shows the contributions of clinical features to each latent variable, were generated. Hierarchical clustering of the H matrix revealed distinct patterns among the clinical features. The heatmap visualization of the reordered H matrix showed distinct patterns, with four main clusters corresponding to the four NMF modules. These modules were designated as “Module QOL,” “Module Diplopia,” “Module Ptosis,” and “Module Systemic,” based on the clinical features in each cluster (Fig 2). The clustering results demonstrated that NMF effectively captured the underlying structure of the data, with clear separations observed across features. Comparing the W matrix between the non-MM and MM or better groups revealed that all Module scores were significantly lower in the MM or better group (Fig 3), similar to the original MG clinical score.

### Model training and cross-validation

**Fig 4:**
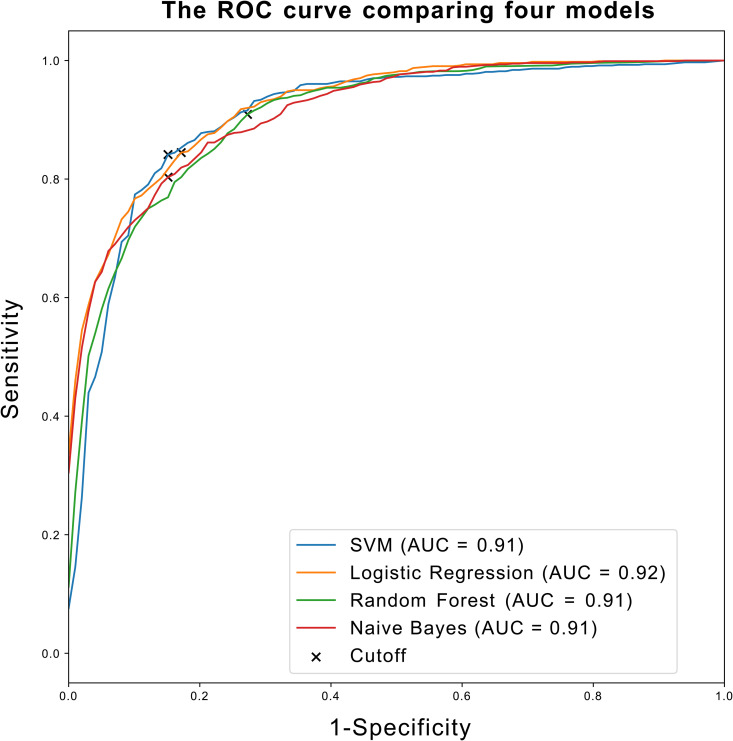
The Receiver Operating Characteristic (ROC) curves comparing the predictive performance of four models on the test dataset. The ROC curves for each model are shown, with corresponding the area under the curve (AUC) presented in the legend. The colors of the lines represent different models: SVM (blue), logistic regression (yellow), random forest (green), and naïve Bayes (red). The optimal cutoff point for each model is marked with a black “×”. The dashed diagonal line represents the performance of a random classifier (AUC = 0.5).

Using the W matrix from NMF, four machine learning models—SVM, Logistic Regression, Random Forest, and Naive Bayes—were trained to predict whether patients had achieved a state of MM or better. Hyperparameter tuning via GridSearchCV improved the performance of all models. After tuning, the Logistic Regression model achieved the highest AUROC score among the models (Fig 4, S1 and S2 Figs).

### Evaluation of ensemble learning model

**Fig 5:**
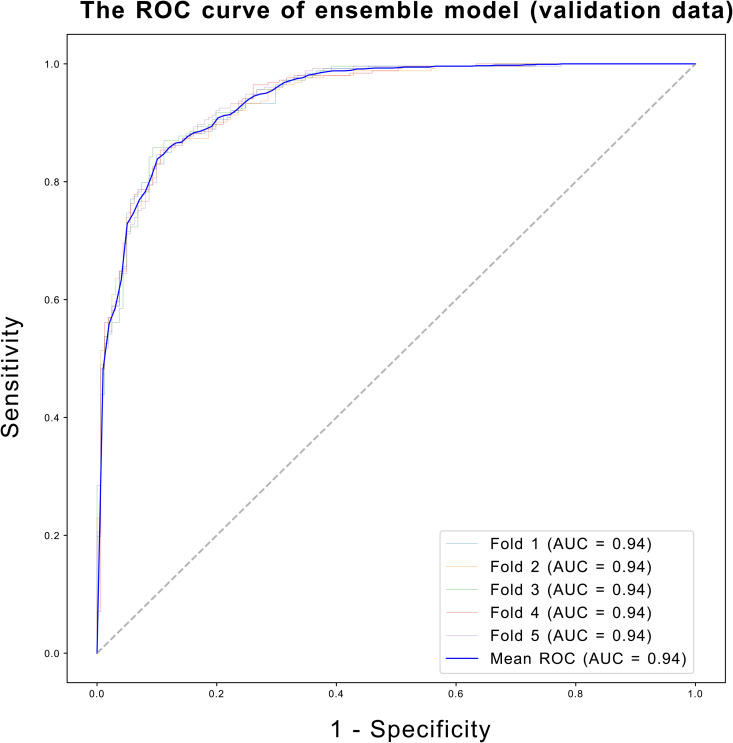
The Receiver Operating Characteristic (ROC) curve illustrating the performance of the ensemble model on the validation dataset. The ROC curve (blue line) represents the relationship between sensitivity and 1-specificity at various threshold settings. The area under the curve (AUC) is shown in the lower right. The dashed diagonal line represents the performance of a random classifier (AUC = 0.5).

**Fig 6:**
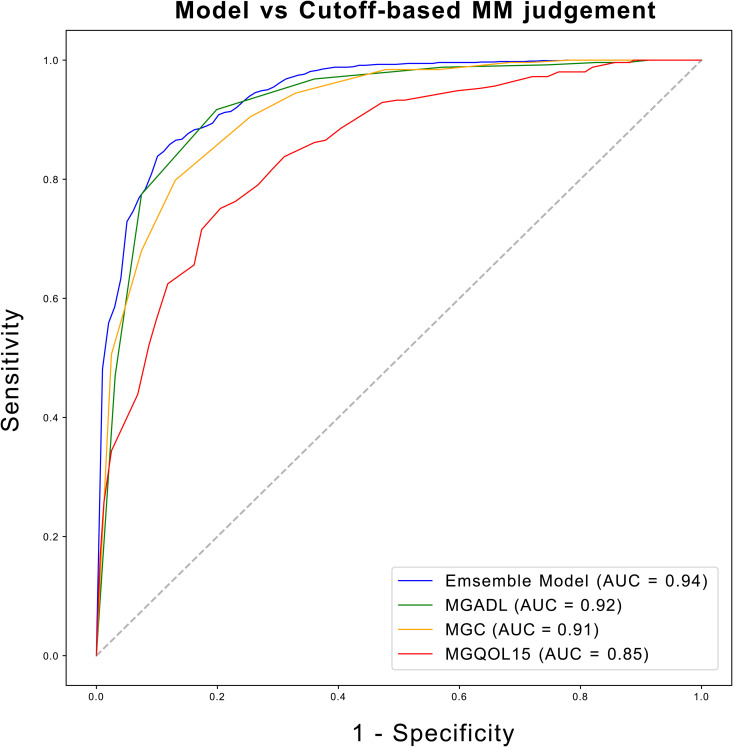
Comparison of ROC curves between the ensemble model and cutoff approaches for MM or better status prediction. The ROC curves compare the performance of our ensemble machine learning model against traditional rule-based approaches using individual clinical scores. The ensemble model (blue line) achieved an AUC of 0.94, demonstrating superior discriminative ability compared to rule-based methods using MGADL alone (AUC = 0.92, green line), MGC alone (AUC = 0.91, orange line), and MGQOL15 alone (AUC = 0.85, red line). The diagonal dashed line represents the performance of a random classifier (AUC = 0.5).

The majority voting ensemble, which combined predictions from all four models, resulted in a further improvement in prediction performance. The ensemble model achieved an AUROC of 0.96 across the cross-validation folds, outperforming any individual model (S3 Fig). This demonstrated the strength of ensemble learning in aggregating the diverse strengths of the individual classifiers, leading to a more accurate and robust prediction. The generalization ability of the ensemble model was further evaluated using an independent dataset of 414 patients who had participated in the 2015 survey. The ensemble model achieved an AUROC of 0.94 (95% CI: 0.94–0.94), accuracy of 0.87 (95% CI: 0.86–0.88), sensitivity of 0.85 (95% CI: 0.85–0.86), specificity of 0.89 (95% CI: 0.88–0.91), precision of 0.93 (95% CI: 0.92–0.94), F1 score of 0.89 (95% CI: 0.88–0.89), and MCC of 0.74 (95% CI: 0.72–0.75) on the validation dataset, confirming its strong generalization performance (Fig 5). Furthermore, the ensemble model outperformed models based on individual clinical scores in predicting MM or better status, achieving an AUC of 0.94, compared to 0.92 for MGADL, 0.91 for MGC, and 0.85 for MGQOL15 (Fig 6). The baseline characteristics of the validation cohort are shown in S1 Table. The validation cohort demonstrated similar demographic and clinical patterns to the training dataset, with comparable distributions of MG subtypes and clinical severity scores. The consistently high AUROC values indicates that the model can reliably distinguish between patients who have achieved MM or better and those who have not, even when applied to a temporally independent dataset.

## Discussion

Assessing MG symptoms often relies on subjective patient reports and clinical judgment. Therefore, the appropriateness and validity of these clinical scores may be subject to personal bias, especially for physicians unfamiliar with MG practice. Even though clinical scores are applied as treatment benchmarks, the choice of suitable scores and the determination of definitive cutoff values still leave room for further discussion. Research on thresholds for MG clinical scores using the PASS (Patient-acceptable symptom state) have indicated that patients feel themselves to be in an acceptable state if they score within 0–2 on the MGADL scale, within 3 on the MGC, and within 8 on the MGQOL15 questionnaire [15]. Also, MGADL scores of 3 or higher were determined to be unsatisfactory status in a Swedish cohort study [16]. However, in clinical practice, we often find that it is not possible to reliably predict a state of MM or better using single score thresholds. For instance, there are cases where the MGADL score is below the threshold while the MGQOL15r score exceeds it, or the opposite can also occur. Additionally, especially with MGADL, using a simple clinical score cutoff can result in patients exceeding the threshold based solely on ocular symptoms. In other words, the cutoff for each clinical score, or the determination of MM or better based on a combination of scores, can often be inconsistent.

In our study, a large dataset containing 1603 cases assessed by physicians with expertise in MG management was analyzed. We employed NMF as an effective technique for understanding the relationships across our cases and various score parameters. NMF is a matrix decomposition method that decomposes our original matrix (V) of cases and score parameters into the product of two non-negative matrices (W and H), following the formula V ≈ WH. The resulting decomposition, represented by V ≈ WH, provides a concise and easily interpretable representation of the data and revealed potential structures within the data set. We have showed that MG clinical scores can potentially be categorized into Ptosis, Diplopia, Systemic symptoms, and Quality of Life components, and utilized these modules as features in machine learning to develop a model that integrates the patient’s clinical scores to determine if treatment goals; MM or better, have been achieved. Our model breaks down and visualizes the patient’s condition into four simple modules to support the physician’s judgment and also provides a prediction of MM or better based on our cohort.

The absence of established biomarkers in the clinical evaluation of MG presents a challenge in determining treatment effectiveness in clinical trials and daily clinical practice. Present assessments mainly rely on individual physician judgment. Implementing a model such as the one proposed would allow for referencing a shared evaluative standard, potentially simplifying the assessment process and fostering more consistent outcomes globally. Recent advancements in the treatment of MG have been remarkable. This progress has been marked by the development of a range of molecularly targeted drugs, including therapies against complement, neonatal FcRn, and CD20 [17]. By setting unified MM or better clinical decisions as the treatment objectives, we can guide the selection of these novel therapies for suitable patients, thus further improving patient outcomes.

This study has several limitations which should be acknowledged. It is essential to ensure that the criteria used in this model rely on reliable data since their accuracy depends on the data quality used in the training process. Also, this data reflects the practice of MG in Japan, as it was determined by physicians familiar with the practice of MG at multiple facilities in Japan. Our model may not be easily adapted as-is for different countries and regions, and the model might need to be retrained.

## Conclusions

This study has presented a machine-learning model that provides a comprehensive and uniform method for assessing treatment success in MG patients. This model can reduce personal bias and promote more consistent treatment outcomes by utilizing dimensionality reduction techniques and integrating various clinical scores. As research and treatment for MG continue to advance, incorporating decision aids like this diagnostic model becomes increasingly important.

## Supporting information

S1 Fig ROC curves for machine learning model performance on training data. ROC curves and AUC values for four machine learning models evaluated on training data using 5-fold cross-validation. (a) Logistic Regression, (b) Naive Bayes, (c) Random Forest, (d) SVM. Individual fold ROC curves (colored lines) and mean ROC curve (thick line) are shown with corresponding AUC values.
(PDF)

S2 Fig ROC curves for machine learning model performance on test data. ROC curves and AUC values for four machine learning models evaluated on test data using 5-fold cross-validation. (a) Logistic Regression, (b) Naive Bayes, (c) Random Forest, (d) SVM. Individual fold ROC curves (colored lines) and mean ROC curve (thick line) are shown with corresponding AUC values.
(PDF)

S3 Fig ROC curves for machine learning model performance on validation data. ROC curves and AUC values for four machine learning models evaluated on validation data using 5-fold cross-validation. (a) Logistic Regression, (b) Naive Bayes, (c) Random Forest, (d) SVM. Individual fold ROC curves (colored lines) and mean ROC curve (thick line) are shown with corresponding AUC values.
(PDF)

S1 Table The profiles of 414 MG patients in validation data. (CSV)
