# Supplementary figures and images for: Predicting achievement of clinical goals using machine learning in myasthenia gravis
Source: PLoS One. 2025 Aug 14;20(8):e0330044. doi: 10.1371/journal.pone.0330044 (PMC12352761; doi:10.1371/journal.pone.0330044)

# S1 Figure

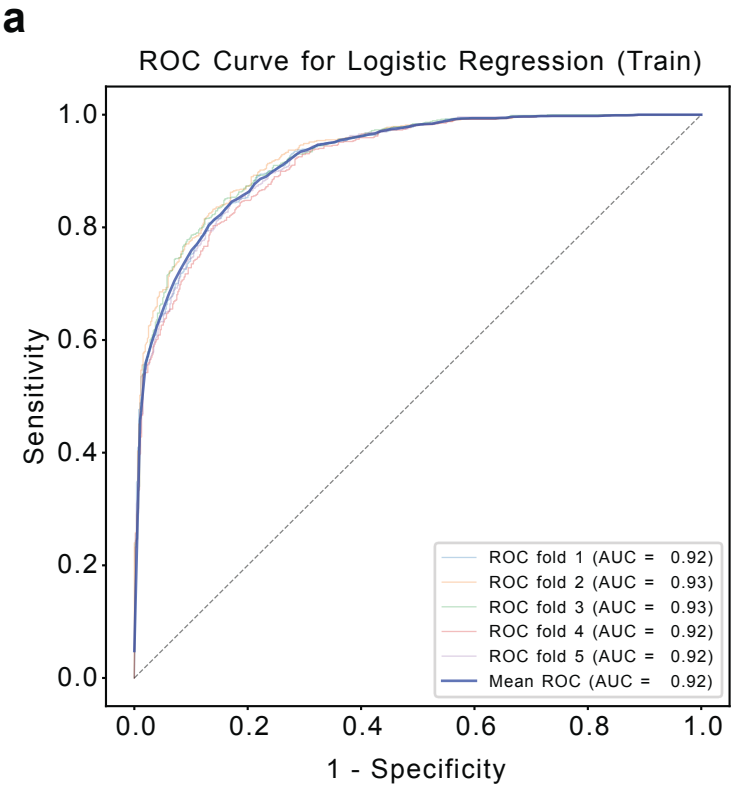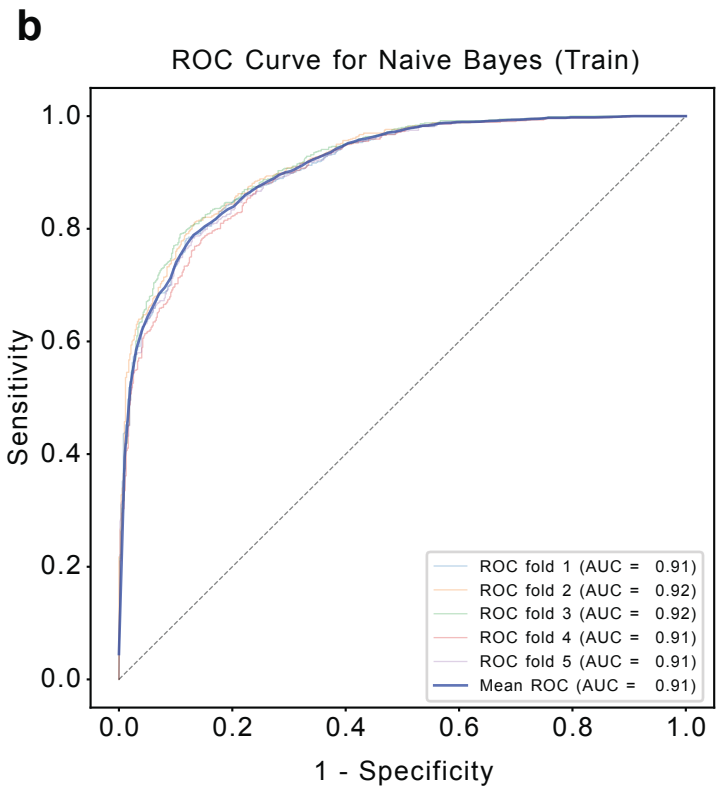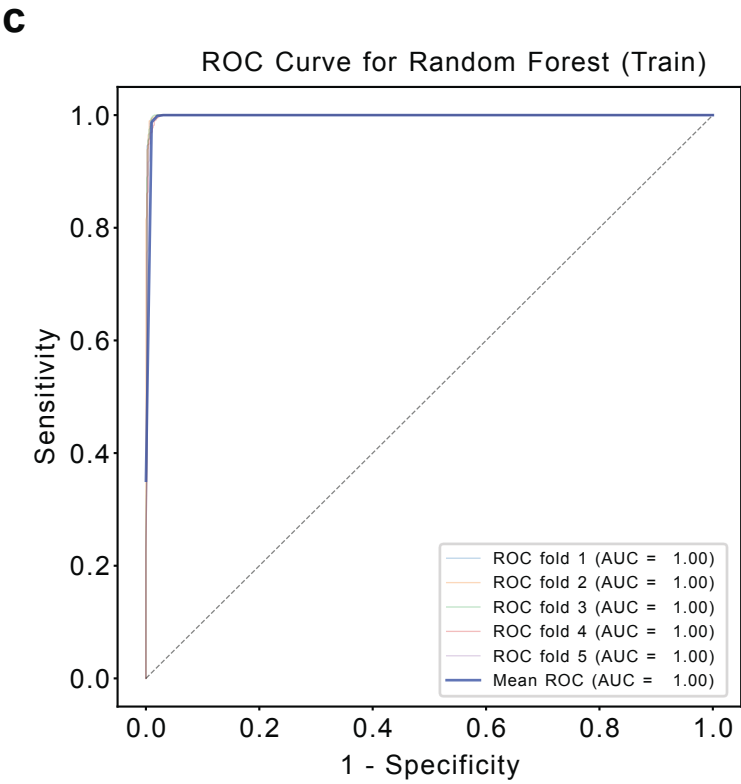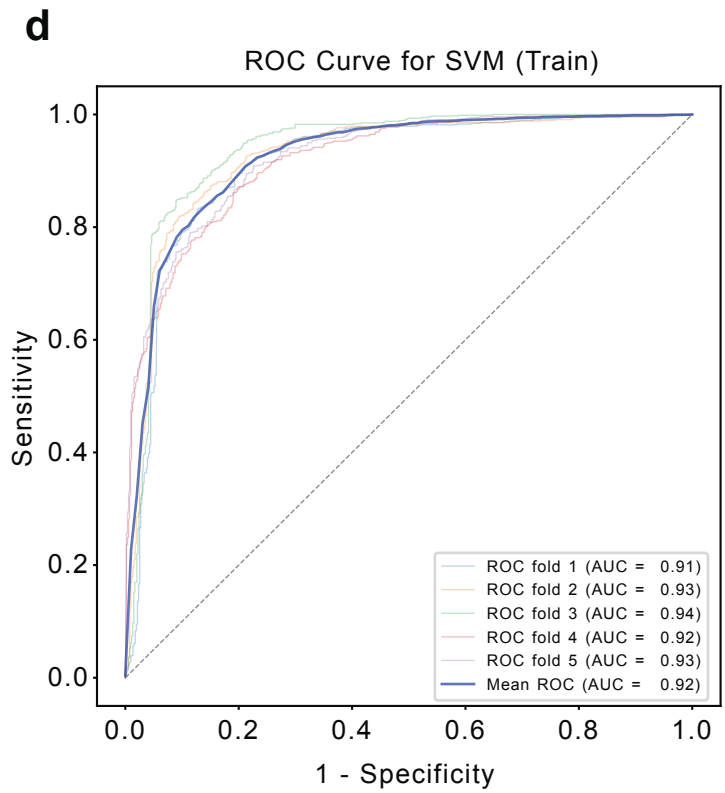

Supplement: S1 Fig — ROC curves and AUC values for four machine learning models evaluated on training data using 5-fold cross-validation. (a) Logistic Regression, (b) Naive Bayes, (c) Random Forest, (d) SVM. Individual fold ROC curves (colored lines) and mean ROC curve (thick line) are shown with corresponding AUC values. (PDF) [file pone.0330044.s001.pdf]

# S2 Figure

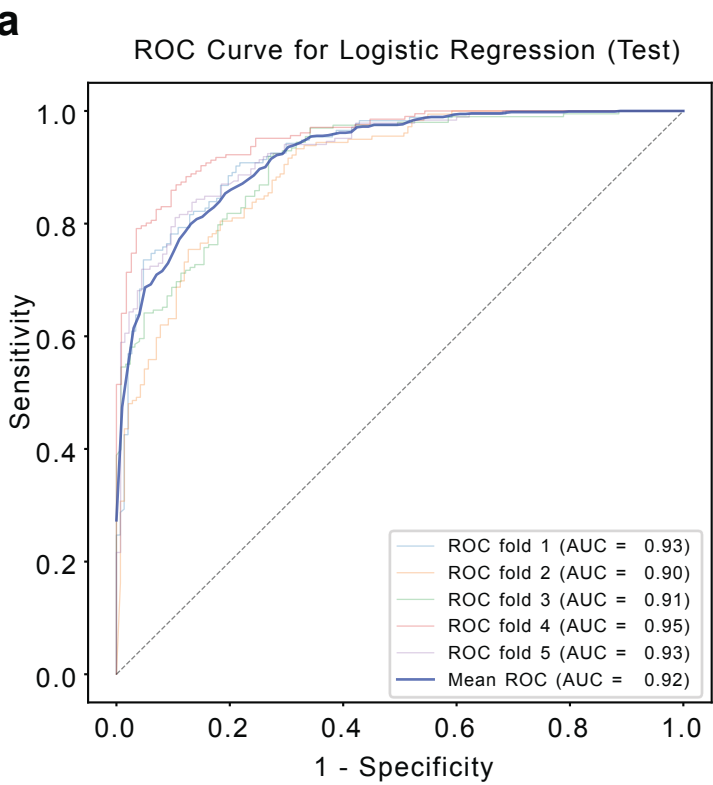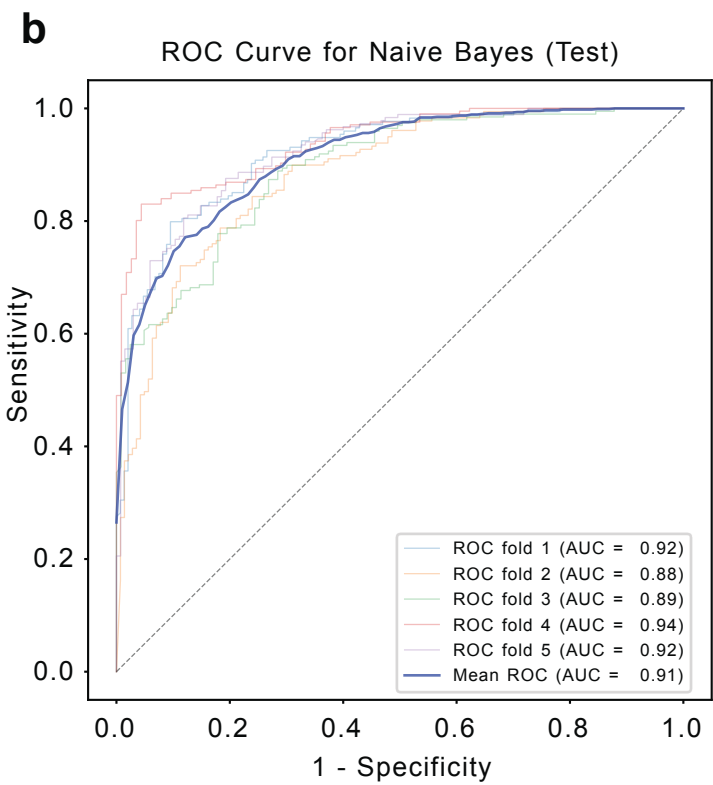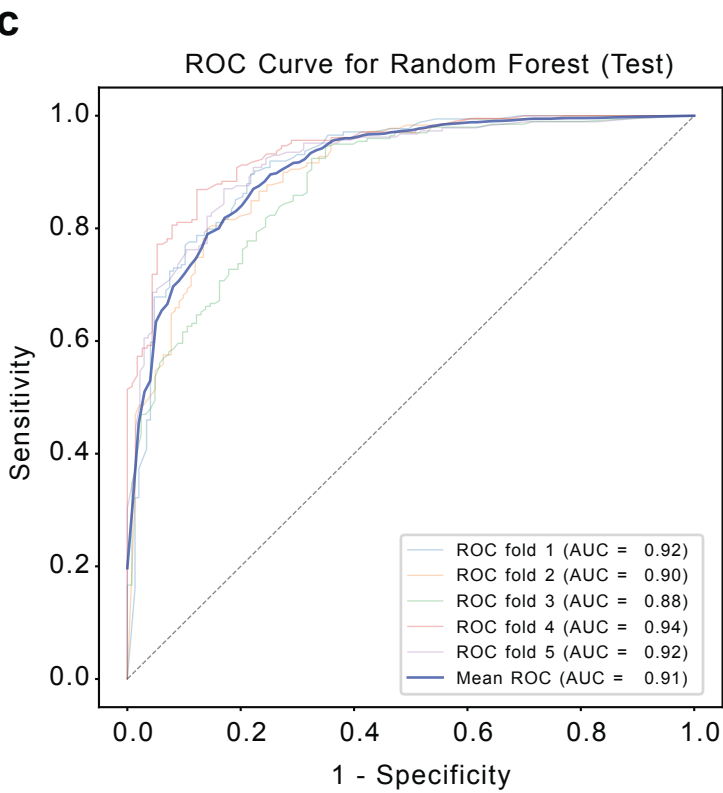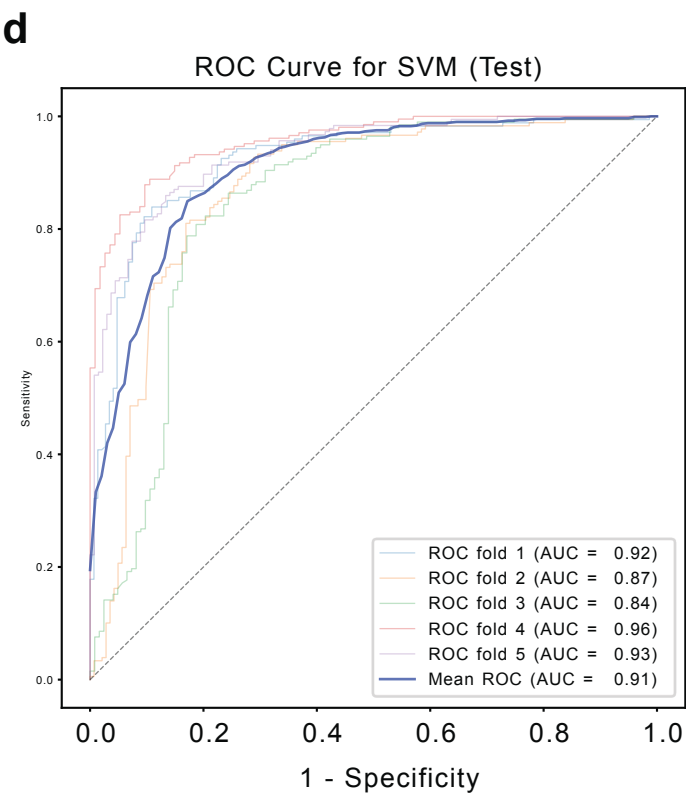

Supplement: S2 Fig — ROC curves and AUC values for four machine learning models evaluated on test data using 5-fold cross-validation. (a) Logistic Regression, (b) Naive Bayes, (c) Random Forest, (d) SVM. Individual fold ROC curves (colored lines) and mean ROC curve (thick line) are shown with corresponding AUC values. (PDF) [file pone.0330044.s002.pdf]

# S3 Figure

**a**

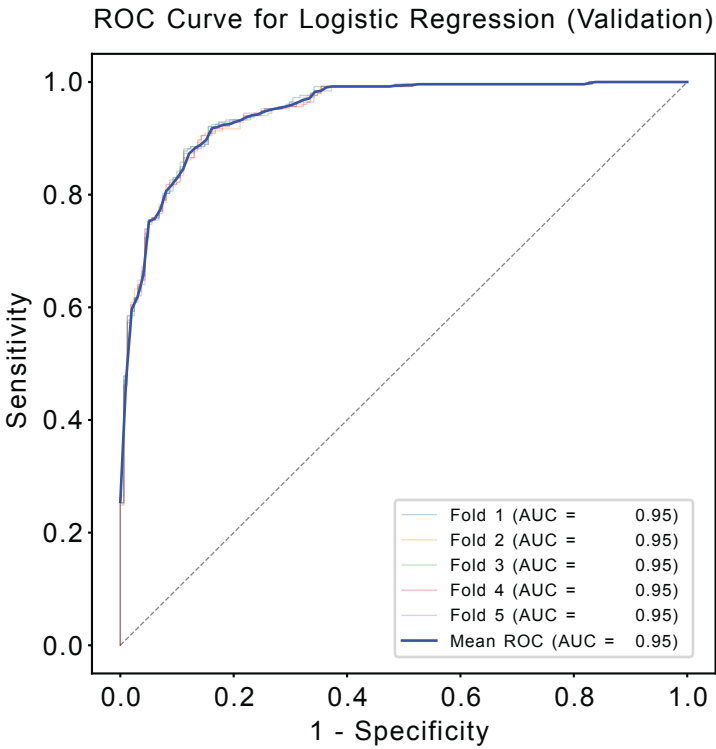

**b**

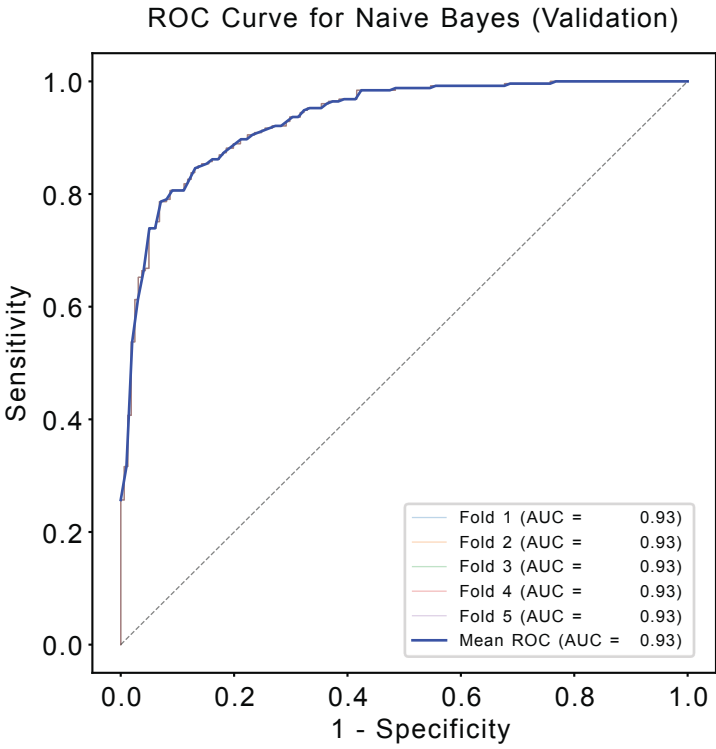

**c**

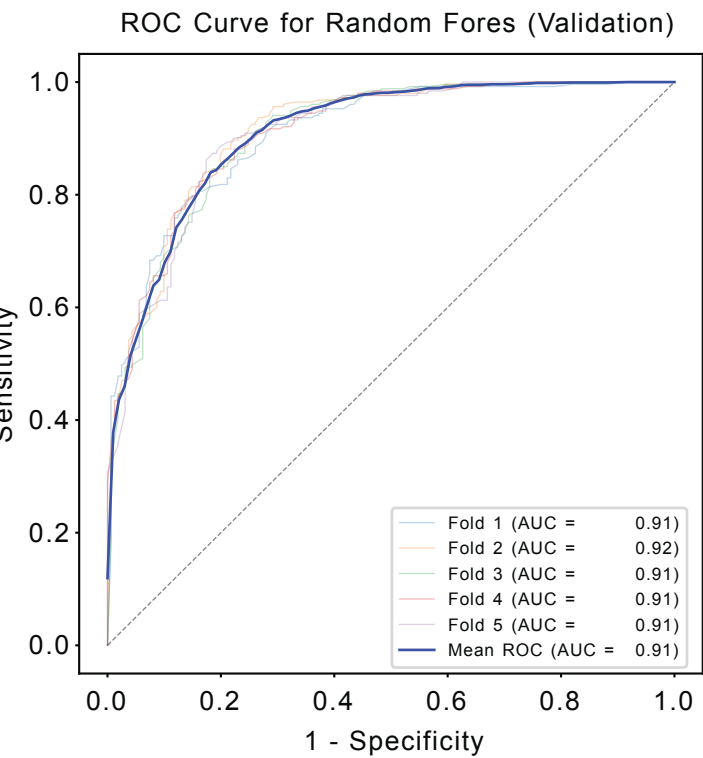

**d**

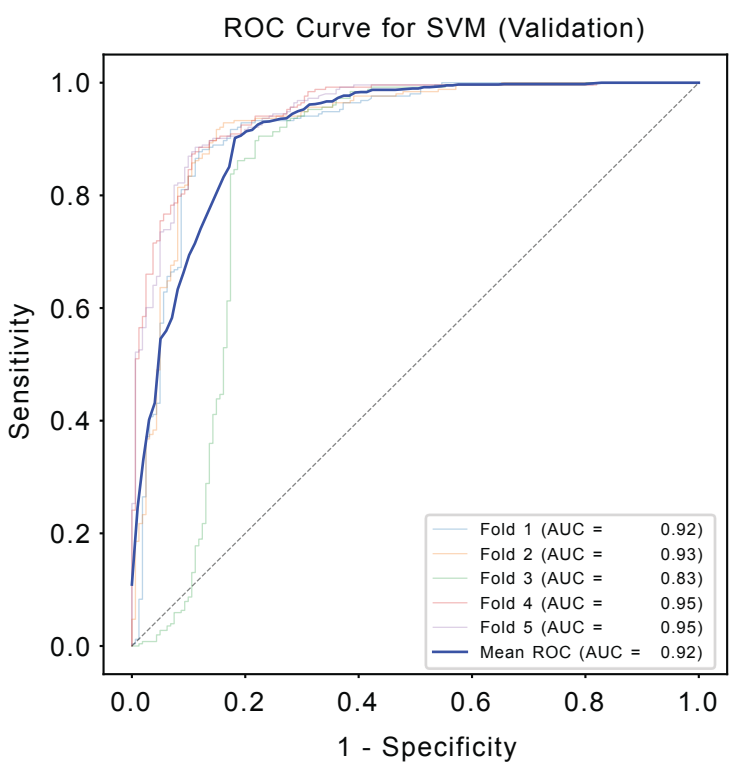

Supplement: S3 Fig — ROC curves and AUC values for four machine learning models evaluated on validation data using 5-fold cross-validation. (a) Logistic Regression, (b) Naive Bayes, (c) Random Forest, (d) SVM. Individual fold ROC curves (colored lines) and mean ROC curve (thick line) are shown with corresponding AUC values. (PDF) [file pone.0330044.s003.pdf]
