# Supplementary material for: Predicting achievement of clinical goals using machine learning in myasthenia gravis
Source: PLoS One. 2025 Aug 14;20(8):e0330044. doi: 10.1371/journal.pone.0330044 (PMC12352761; doi:10.1371/journal.pone.0330044)
Supplement: Attachment — Submitted filename: Response to Reviewers.docx [file pone.0330044.s006.docx]

**Response to Reviewers**

**Reviewer #1:**

Comment #1
The introduction effectively outlines the pathophysiology, classification, and assessment tools for Myasthenia Gravis (MG), making it informative for readers. Highlighting the challenges in defining Minimal Manifestation (MM) status aligns well with the study's objective of standardization, making the research question clear. Also, the transition from general MG characteristics to the specific issue of MM definition is well-structured.

Response:
We thank the reviewer for these positive comments about our introduction and the presentation of the research question. In the introduction section, we described the pathophysiology of MG, outlined the various clinical assessment tools (QMG, MGADL, MGC, and MGQOL15r), and highlighted the challenges in MM status determination.

Comment #2
"However, there is no clear definition to identify minimal muscle weakness that is clinically important, which can result in variability in how physicians interpret and apply the MM criteria." Comment: The variability in MM criteria is a key gap, but the impact of this inconsistency on clinical decision-making should be more explicitly stated. Does it lead to differences in treatment approaches, misclassification of patients, or difficulties in research comparability?

Response:
We thank the reviewer for this important and insightful comment. We completely agree that the clinical impact of MM criteria variability should be more clearly described, as this is fundamental to understanding the significance of our research. We have revised the manuscript to better explain how this inconsistency may affect clinical practice.

*Revised text (lines 121-128):
"There is no clear definition to identify minimal muscle weakness that is clinically important, which can result in variability in how physicians interpret and apply the MM criteria. This inconsistency may lead to different treatment approaches and creates challenges in comparing outcomes across medical centers. By providing visualization of MG symptoms in MM determination and standardizing the assessment process, our approach aims to support more unified treatment selection criteria and help identify unmet clinical needs in MG management."*

Comment #3
"The quantitative MG (QMG) scale, the MG activities of daily living (MGADL) score, the MG composite (MGC) scale are widely used to evaluate the clinical symptoms of MG patients." Comment: The sentence is dense and would benefit from breaking down each scale's purpose briefly (e.g., QMG for objective severity, MGADL for daily function, MGC as a composite measure).

Response:
We thank the reviewer for this helpful and constructive suggestion. We have revised the text to clearly describe the distinct roles of these clinical assessment tools.

*Revised text (line 106-111):
"Several standardized scales are widely used to evaluate the clinical symptoms of MG patients: the quantitative MG (QMG) scale for objective assessment of muscle weakness severity, the MG activities of daily living (MGADL) score for evaluating functional limitations in daily activities, and the MG composite (MGC) scale as a comprehensive measure combining both objective and subjective assessments."*

Comment #4
Use "Myasthenia Gravis (MG)" at first mention, then "MG" thereafter for consistency.

Response:
We thank the reviewer for pointing out this important inconsistency in our terminology usage. We have carefully revised the entire manuscript to ensure consistent terminology throughout, using "Myasthenia Gravis (MG)" at the first mention and "MG" thereafter.

**Reviewer #2:**

Comment #1
Throughout the results (AUROC, sensitivity, specificity) results are given without 95% CI. I think it would be valuable to assess the variation in these results based on the 95% CI range and the authors should include them for each result, both in the abstract and the main results.

Response:
We thank the reviewer for this excellent and important suggestion. We agree that reporting 95% confidence intervals is essential for properly assessing the reliability and precision of our results. We have comprehensively revised the manuscript to include 95% confidence intervals calculated from the 5-fold cross-validation results for AUC values, and comprehensive metrics with 95% CIs for the final ensemble model validation results throughout both the abstract and main results sections.

Comment #2
The authors state that the clinical status was assessed by physicians expert in MG treatment- were these physicians primary care, rheumatologists, or any other?

Response:
We thank the reviewer for this important clarification request, as the expertise level of assessing physicians is crucial information for evaluating the validity of our clinical assessments. The clinical status assessments were conducted by neurologists with extensive experience in MG diagnosis and management at specialized neuromuscular centers across Japan. We have added this clarification to the main text in the Data Collection and Access section (lines 139-140), and in the Data Processing section (lines 145-148).

*Added text: line 139-140
“The clinical assessments were performed by neurologists with extensive experience in MG diagnosis and management.”*

*Added text: line 145-148
“The evaluation of these clinical scores, as well as the determination of whether a patient had reached MM or better status (MM, Complete Stable Remission, or Pharmacologic Remission), was conducted by neurologists with expertise in MG management.”*

Comment #3
What were the baseline characteristics of patients in the 2015 validation cohort? A table summarizing them, perhaps in the supplement, will enhance the findings in the study.

Response:
We thank the reviewer for this valuable and insightful suggestion. We have added a comprehensive supplementary table (Supplementary Table S1) summarizing the demographic and clinical characteristics of the validation dataset.

*Added text: line 293-296
"The baseline characteristics of the validation cohort are presented in Supplementary Table S1. The validation cohort demonstrated similar demographic and clinical patterns to the training dataset, with comparable distributions of MG subtypes and clinical severity scores."*

**Reviewer #3:**

Comment #1
The authors should provide a thorough review of existing models that predict MM status in MG patients.

Response:
We thank the reviewer for this important suggestion. After conducting a comprehensive literature review, we found that, to our knowledge, no previous studies have developed machine learning models specifically for MM status prediction in MG patients. This represents a significant gap in the literature that our study addresses.

Comment #2
If other models for MM prediction exist, the authors need to clearly highlight the advantages of their approach in comparison.

Response:
We thank the reviewer for this excellent point. While no machine learning models exist specifically for MM prediction, existing clinical approaches rely on single clinical score cutoffs, which have inherent limitations. To demonstrate the superiority of our approach, we compared our ensemble model performance with individual score cutoff methods in the validation dataset. Our ensemble model achieved an AUC of 0.94, demonstrating clear superiority over rule-based methods using individual scores: MGADL alone (AUC = 0.92), MGC alone (AUC = 0.91), and MGQOL15 alone (AUC = 0.85). This comparison clearly demonstrates the advantage of our integrated machine learning approach over traditional single-score cutoff methods (Fig 6).

Comment #3
(Line 249): The legend for Figure 2 appears after the legend for Figure 3. Please correct the order.

Response:
We thank the reviewer for identifying this formatting error. We have carefully corrected the figure legend order throughout the manuscript to ensure proper sequential numbering and presentation.

Comment #4
(Line 250): Change "he H matrix" to "The H matrix".

Response:
We thank the reviewer for catching this typographical error. This has been corrected.

Comment #5
(Line 261): The authors should report both training and test results for all four models, including SVM, logistic regression, random forest, and Naive Bayes.

Response:
We thank the reviewer for this important suggestion. We completely agree that comprehensive reporting of both training and test results for all models enhances the transparency and completeness of our evaluation. We have added detailed ROC curves showing both training and test results for all four individual models (SVM, logistic regression, random forest, and Naive Bayes) in the supplementary figures (S1-S3 Figures).

Comment #6
(Line 261): Since a 5-fold cross-validation was used, the variability across the five folds should be reported.

Response:
We thank the reviewer for this excellent point about reporting cross-validation variability. We have comprehensively reported the variability across all five cross-validation folds by showing individual fold results in our ROC curve figures (Supplementary Figures 1-3). Given the manageable number of folds (n=5), we present all individual AUC values for each fold rather than just summary statistics, providing complete transparency about model performance variability.

Comment #7
(Line 274): Similarly, for the ensemble model, both training and test performance should be shown, along with the variability across the five cross-validation folds.

Response:
We thank the reviewer for this important suggestion. We have included comprehensive results showing both training and test performance for the ensemble model, with individual fold results displayed in the ROC curves to demonstrate the complete range of performance across all five cross-validation iterations (Fig 5).

Comment #8
(Line 277): Given that five iterations were conducted, was an ensemble model generated for each iteration? If so, how were the five ensemble models applied to the external validation dataset? Please clarify.

Response:
We thank the reviewer for requesting this important methodological clarification. For external validation, we used all five trained models from each cross-validation fold: for each validation sample, we calculated predictions from all five folds and used soft voting (averaging probabilities) to generate the final ensemble prediction.

*line 193-195:
“For performance evaluation including AUROC, we applied a soft voting strategy, averaging the predicted probabilities from all four models to generate the final prediction scores.”*

Comment #9
Figures 4 and 5: The ROC curves shown in Figures 4 and 5 use inconsistent font sizes, legend locations, and titles. Please ensure uniform formatting.

Response:
We thank the reviewer for this attention to detail regarding figure presentation. We have carefully reviewed and uniformly corrected the formatting of all ROC curve figures, ensuring consistent font sizes, legend locations, and titles throughout the manuscript.
