# Supplementary material for: Predicting achievement of clinical goals using machine learning in myasthenia gravis
Source: PLoS One. 2025 Aug 14;20(8):e0330044. doi: 10.1371/journal.pone.0330044 (PMC12352761; doi:10.1371/journal.pone.0330044)
Supplement: Peer review history

# Peer review: Decision Letter 0

**DOI:** 10.1371/journal.pone.0330044.r001

**Authors:** Pinyi Lu

29 Apr 2025

Dear Dr. Uzawa,

Thank you for submitting your manuscript to PLOS ONE. After careful consideration, we feel that it has merit but does not fully meet PLOS ONE’s publication criteria as it currently stands. Therefore, we invite you to submit a revised version of the manuscript that addresses the points raised during the review process.

Please submit your revised manuscript by Jun 13 2025 11:59PM. If you will need more time than this to complete your revisions, please reply to this message or contact the journal office at plosone@plos.org . When you're ready to submit your revision, log on to https://www.editorialmanager.com/pone/ and select the 'Submissions Needing Revision' folder to locate your manuscript file.

- A rebuttal letter that responds to each point raised by the academic editor and reviewer(s). You should upload this letter as a separate file labeled 'Response to Reviewers'.
- A marked-up copy of your manuscript that highlights changes made to the original version. You should upload this as a separate file labeled 'Revised Manuscript with Track Changes'.
- An unmarked version of your revised paper without tracked changes. You should upload this as a separate file labeled 'Manuscript'.

If you would like to make changes to your financial disclosure, please include your updated statement in your cover letter. Guidelines for resubmitting your figure files are available below the reviewer comments at the end of this letter.

If applicable, we recommend that you deposit your laboratory protocols in protocols.io to enhance the reproducibility of your results. Protocols.io assigns your protocol its own identifier (DOI) so that it can be cited independently in the future. For instructions see: https://journals.plos.org/plosone/s/submission-guidelines#loc-laboratory-protocols . Additionally, PLOS ONE offers an option for publishing peer-reviewed Lab Protocol articles, which describe protocols hosted on protocols.io. Read more information on sharing protocols at https://plos.org/protocols?utm_medium=editorial-email&utm_source=authorletters&utm_campaign=protocols .

We look forward to receiving your revised manuscript.

Kind regards,

Pinyi Lu, Ph.D.

Academic Editor

PLOS ONE

Journal requirements:

When submitting your revision, we need you to address these additional requirements.

1.Please ensure that your manuscript meets PLOS ONE's style requirements, including those for file naming. The PLOS ONE style templates can be found at

https://journals.plos.org/plosone/s/file?id=wjVg/PLOSOne_formatting_sample_main_body.pdf and

https://journals.plos.org/plosone/s/file?id=ba62/PLOSOne_formatting_sample_title_authors_affiliations.pdf

2. Please note that funding information should not appear in any section or other areas of your manuscript. We will only publish funding information present in the Funding Statement section of the online submission form. Please remove any funding-related text from the manuscript.

3. We note that the grant information you provided in the ‘Funding Information’ and ‘Financial Disclosure’ sections do not match.

When you resubmit, please ensure that you provide the correct grant numbers for the awards you received for your study in the ‘Funding Information’ section.

4. Thank you for stating the following in the Competing Interests section:

“AU has received honoraria from Alexion Pharmaceuticals and Argenx. SS received speakers’ fees from Alexion Pharmaceuticals, the Japan Blood Products Organization, Asahi Kasei Medical and Argenx and participated on advisory board meeting of Alexion Pharmaceuticals and Argenx. SK has received Research Grant on Intractable Diseases (Neuroimmunological Diseases) from the Ministry of Health, Labour and Welfare of Japan (20FC1030) and honoraria from CSL Behring. TK reports honorarium for lectures from Alexon Pharmaceuticals.

MPT reports unrestricted research grants from Japan Blood Products Organization, Astellas Pharma, Mitsubishi Tanabe Pharma and Pfizer, outside the submitted work, and has served as a paid Consultant for Alexion, Argenx, Sanofi, and UCB Pharma and received honorarium for lectures from Argenx, Alexion Pharmaceuticals and UCB Pharma. MM has received speaker honoraria from Argenx, Asahi Kasei Medical, Alexion Pharmaceutical and participated on advisory board meeting of Alexion Pharmaceutical. KU has served as a paid Consultant for UCB Pharma, Janssen Pharma, Horizon Therpeutics (Viela Bio), Chugai Pharma, Hanall BioPharma and Mitsubishi Tanabe Pharma, and has received speaker honoraria from Argenx, Alexion Pharmaceuticals, UCB Pharma, and the Japan Blood Products Organisation. Other authors declare no financial or other conflicts of interest.

YN has received speaker honoraria from argenx, Alexion Pharmaceuticals, Japan Blood Products Organization, and UCB Pharma.”

We note that you received funding from a commercial source: Alexion Pharmaceuticals, Argenx, Japan Blood Products Organization, Astellas Pharma, Mitsubishi Tanabe Pharma, Pfizer and UCB Pharma

Please provide an amended Competing Interests Statement that explicitly states this commercial funder, along with any other relevant declarations relating to employment, consultancy, patents, products in development, marketed products, etc.

Within this Competing Interests Statement, please confirm that this does not alter your adherence to all PLOS ONE policies on sharing data and materials by including the following statement: "This does not alter our adherence to PLOS ONE policies on sharing data and materials.” (as detailed online in our guide for authors http://journals.plos.org/plosone/s/competing-interests).  If there are restrictions on sharing of data and/or materials, please state these. Please note that we cannot proceed with consideration of your article until this information has been declared.

Please include your amended Competing Interests Statement within your cover letter. We will change the online submission form on your behalf.

5. In this instance it seems there may be acceptable restrictions in place that prevent the public sharing of your minimal data. However, in line with our goal of ensuring long-term data availability to all interested researchers, PLOS’ Data Policy states that authors cannot be the sole named individuals responsible for ensuring data access (http://journals.plos.org/plosone/s/data-availability#loc-acceptable-data-sharing-methods).

Data requests to a non-author institutional point of contact, such as a data access or ethics committee, helps guarantee long term stability and availability of data. Providing interested researchers with a durable point of contact ensures data will be accessible even if an author changes email addresses, institutions, or becomes unavailable to answer requests.

Before we proceed with your manuscript, please also provide non-author contact information (phone/email/hyperlink) for a data access committee, ethics committee, or other institutional body to which data requests may be sent. If no institutional body is available to respond to requests for your minimal data, please consider if there any institutional representatives who did not collaborate in the study, and are not listed as authors on the manuscript, who would be able to hold the data and respond to external requests for data access? If so, please provide their contact information (i.e., email address). Please also provide details on how you will ensure persistent or long-term data storage and availability.

6. Your ethics statement should only appear in the Methods section of your manuscript. If your ethics statement is written in any section besides the Methods, please move it to the Methods section and delete it from any other section. Please ensure that your ethics statement is included in your manuscript, as the ethics statement entered into the online submission form will not be published alongside your manuscript.

7. We note that Figure 1 in your submission contain map/satellite images which may be copyrighted. All PLOS content is published under the Creative Commons Attribution License (CC BY 4.0), which means that the manuscript, images, and Supporting Information files will be freely available online, and any third party is permitted to access, download, copy, distribute, and use these materials in any way, even commercially, with proper attribution. For these reasons, we cannot publish previously copyrighted maps or satellite images created using proprietary data, such as Google software (Google Maps, Street View, and Earth). For more information, see our copyright guidelines: http://journals.plos.org/plosone/s/licenses-and-copyright.

 We require you to either (a) present written permission from the copyright holder to publish these figures specifically under the CC BY 4.0 license, or (b) remove the figures from your submission:

 a. You may seek permission from the original copyright holder of Figure 1 to publish the content specifically under the CC BY 4.0 license. 

 We recommend that you contact the original copyright holder with the Content Permission Form (http://journals.plos.org/plosone/s/file?id=7c09/content-permission-form.pdf) and the following text:

“I request permission for the open-access journal PLOS ONE to publish XXX under the Creative Commons Attribution License (CCAL) CC BY 4.0 (http://creativecommons.org/licenses/by/4.0/). Please be aware that this license allows unrestricted use and distribution, even commercially, by third parties. Please reply and provide explicit written permission to publish XXX under a CC BY license and complete the attached form.”

 Please upload the completed Content Permission Form or other proof of granted permissions as an "Other" file with your submission.

 In the figure caption of the copyrighted figure, please include the following text: “Reprinted from [ref] under a CC BY license, with permission from [name of publisher], original copyright [original copyright year].”

 b. If you are unable to obtain permission from the original copyright holder to publish these figures under the CC BY 4.0 license or if the copyright holder’s requirements are incompatible with the CC BY 4.0 license, please either i) remove the figure or ii) supply a replacement figure that complies with the CC BY 4.0 license. Please check copyright information on all replacement figures and update the figure caption with source information. If applicable, please specify in the figure caption text when a figure is similar but not identical to the original image and is therefore for illustrative purposes only.

The following resources for replacing copyrighted map figures may be helpful:

 USGS National Map Viewer (public domain): http://viewer.nationalmap.gov/viewer/

The Gateway to Astronaut Photography of Earth (public domain): http://eol.jsc.nasa.gov/sseop/clickmap/

Maps at the CIA (public domain): https://www.cia.gov/library/publications/the-world-factbook/index.html and https://www.cia.gov/library/publications/cia-maps-publications/index.html

NASA Earth Observatory (public domain): http://earthobservatory.nasa.gov/

Landsat: http://landsat.visibleearth.nasa.gov/

USGS EROS (Earth Resources Observatory and Science (EROS) Center) (public domain): http://eros.usgs.gov/#

Natural Earth (public domain): http://www.naturalearthdata.com/

Reviewers' comments:

Reviewer's Responses to Questions

**Comments to the Author**

1. Is the manuscript technically sound, and do the data support the conclusions?

Reviewer #1: Yes

Reviewer #2: Yes

Reviewer #3: Yes

**********

2. Has the statistical analysis been performed appropriately and rigorously?

Reviewer #1: Yes

Reviewer #2: Yes

Reviewer #3: Yes

**********

3. Have the authors made all data underlying the findings in their manuscript fully available?

The PLOS Data policy

Reviewer #1: Yes

Reviewer #2: Yes

Reviewer #3: Yes

**********

4. Is the manuscript presented in an intelligible fashion and written in standard English?

Reviewer #1: Yes

Reviewer #2: Yes

Reviewer #3: Yes

**********

Reviewer #1: Comment#1

The introduction effectively outlines the pathophysiology, classification, and assessment tools for Myasthenia Gravis (MG), making it informative for readers. Highlighting the challenges in defining Minimal Manifestation (MM) status aligns well with the study's objective of standardization, making the research question clear. Also, the transition from general MG characteristics to the specific issue of MM definition is well-structured.

Comment#2

"However, there is no clear definition to identify minimal muscle weakness that is clinically important, which can result in variability in how physicians interpret and apply the MM criteria."

Comment: The variability in MM criteria is a key gap, but the impact of this inconsistency on clinical decision-making should be more explicitly stated. Does it lead to differences in treatment approaches, misclassification of patients, or difficulties in research comparability?

Comment#3

"The quantitative MG (QMG) scale, the MG activities of daily living (MGADL) score, the MG composite (MGC) scale are widely used to evaluate the clinical symptoms of MG patients."

Comment: The sentence is dense and would benefit from breaking down each scale's purpose briefly (e.g., QMG for objective severity, MGADL for daily function, MGC as a composite measure).

Comment#4

Use "Myasthenia Gravis (MG)" at first mention, then "MG" thereafter for consistency.

Reviewer #2: This study developed and assessed machine learning models to predict clinical goal achievement in myasthenia gravis. I commend the authors for utilizing artificial intelligence to help monitor management in this patient population. Some comments for consideration:

1. Throughout the results (AUROC, sensitivity, specificity) results are given without 95% CI. I think it would be valuable to assess the variation in these results based on the 95% CI range and the authors should include them for each result, both in the abstract and the main results.

2. The authors state that the clinical status was assessed by physicians expert in MG treatment- were these physicians primary care, rheumatologists, or any other?

3. What were the baseline characteristics of patients in the 2015 validation cohort? A table summarizing them, perhaps in the supplement, will enhance the findings in the study.

Reviewer #3: This manuscript presents a diagnostic model that predicts NM or better status in MG patients. The model achieves good performance on both the training and validation datasets. However, several points need to be addressed before the manuscript can be considered for publication:

1. The authors should provide a thorough review of existing models that predict NM status in MG patients.

2. If other models for NM prediction exist, the authors need to clearly highlight the advantages of their approach in comparison.

3. (Line 249): The legend for Figure 2 appears after the legend for Figure 3. Please correct the order.

4. (Line 250): Change "he H matrix" to "The H matrix".

5. (Line 261): The authors should report both training and test results for all four models, including SVM, logistic regression, random forest, and Naive Bayes.

6. (Line 261): Since a 5-fold cross-validation was used, the variability across the five folds should be reported.

7. (Line 274): Similarly, for the ensemble model, both training and test performance should be shown, along with the variability across the five cross-validation folds.

8. (Line 277): Given that five iterations were conducted, was an ensemble model generated for each iteration? If so, how were the five ensemble models applied to the external validation dataset? Please clarify.

9. Figures 4 and 5: The ROC curves shown in Figures 4 and 5 use inconsistent font sizes, legend locations, and titles. Please ensure uniform formatting.

**********

what does this mean? ). If published, this will include your full peer review and any attached files.

If you choose “no”, your identity will remain anonymous but your review may still be made public.

**Do you want your identity to be public for this peer review?** For information about this choice, including consent withdrawal, please see our Privacy Policy

Reviewer #1: **Yes: ** Ahmed Kamal Siddiqi

Reviewer #2: No

Reviewer #3: No

**********

[NOTE: If reviewer comments were submitted as an attachment file, they will be attached to this email and accessible via the submission site. Please log into your account, locate the manuscript record, and check for the action link "View Attachments". If this link does not appear, there are no attachment files.]

While revising your submission, please upload your figure files to the Preflight Analysis and Conversion Engine (PACE) digital diagnostic tool, https://pacev2.apexcovantage.com/ . PACE helps ensure that figures meet PLOS requirements. To use PACE, you must first register as a user. Registration is free. Then, login and navigate to the UPLOAD tab, where you will find detailed instructions on how to use the tool. If you encounter any issues or have any questions when using PACE, please email PLOS at figures@plos.org . Please note that Supporting Information files do not need this step.

# Author comment: Author response to Decision Letter 1

**DOI:** 10.1371/journal.pone.0330044.r002

3 Jul 2025

Response to Reviewers

Reviewer #1:

Comment #1

The introduction effectively outlines the pathophysiology, classification, and assessment tools for Myasthenia Gravis (MG), making it informative for readers. Highlighting the challenges in defining Minimal Manifestation (MM) status aligns well with the study's objective of standardization, making the research question clear. Also, the transition from general MG characteristics to the specific issue of MM definition is well-structured.

Response:

We thank the reviewer for these positive comments about our introduction and the presentation of the research question. In the introduction section, we described the pathophysiology of MG, outlined the various clinical assessment tools (QMG, MGADL, MGC, and MGQOL15r), and highlighted the challenges in MM status determination.

Comment #2

"However, there is no clear definition to identify minimal muscle weakness that is clinically important, which can result in variability in how physicians interpret and apply the MM criteria." Comment: The variability in MM criteria is a key gap, but the impact of this inconsistency on clinical decision-making should be more explicitly stated. Does it lead to differences in treatment approaches, misclassification of patients, or difficulties in research comparability?

Response:

We thank the reviewer for this important and insightful comment. We completely agree that the clinical impact of MM criteria variability should be more clearly described, as this is fundamental to understanding the significance of our research. We have revised the manuscript to better explain how this inconsistency may affect clinical practice.

Revised text (lines 121-128):

"There is no clear definition to identify minimal muscle weakness that is clinically important, which can result in variability in how physicians interpret and apply the MM criteria. This inconsistency may lead to different treatment approaches and creates challenges in comparing outcomes across medical centers. By providing visualization of MG symptoms in MM determination and standardizing the assessment process, our approach aims to support more unified treatment selection criteria and help identify unmet clinical needs in MG management."

Comment #3

"The quantitative MG (QMG) scale, the MG activities of daily living (MGADL) score, the MG composite (MGC) scale are widely used to evaluate the clinical symptoms of MG patients." Comment: The sentence is dense and would benefit from breaking down each scale's purpose briefly (e.g., QMG for objective severity, MGADL for daily function, MGC as a composite measure).

Response:

We thank the reviewer for this helpful and constructive suggestion. We have revised the text to clearly describe the distinct roles of these clinical assessment tools.

Revised text (line 106-111):

"Several standardized scales are widely used to evaluate the clinical symptoms of MG patients: the quantitative MG (QMG) scale for objective assessment of muscle weakness severity, the MG activities of daily living (MGADL) score for evaluating functional limitations in daily activities, and the MG composite (MGC) scale as a comprehensive measure combining both objective and subjective assessments."

Comment #4

Use "Myasthenia Gravis (MG)" at first mention, then "MG" thereafter for consistency.

Response:

We thank the reviewer for pointing out this important inconsistency in our terminology usage. We have carefully revised the entire manuscript to ensure consistent terminology throughout, using "Myasthenia Gravis (MG)" at the first mention and "MG" thereafter.

Reviewer #2:

Comment #1

Throughout the results (AUROC, sensitivity, specificity) results are given without 95% CI. I think it would be valuable to assess the variation in these results based on the 95% CI range and the authors should include them for each result, both in the abstract and the main results.

Response:

We thank the reviewer for this excellent and important suggestion. We agree that reporting 95% confidence intervals is essential for properly assessing the reliability and precision of our results. We have comprehensively revised the manuscript to include 95% confidence intervals calculated from the 5-fold cross-validation results for AUC values, and comprehensive metrics with 95% CIs for the final ensemble model validation results throughout both the abstract and main results sections.

Comment #2

The authors state that the clinical status was assessed by physicians expert in MG treatment- were these physicians primary care, rheumatologists, or any other?

Response:

We thank the reviewer for this important clarification request, as the expertise level of assessing physicians is crucial information for evaluating the validity of our clinical assessments. The clinical status assessments were conducted by neurologists with extensive experience in MG diagnosis and management at specialized neuromuscular centers across Japan. We have added this clarification to the main text in the Data Collection and Access section (lines 139-140), and in the Data Processing section (lines 145-148).

Added text: line 139-140

“The clinical assessments were performed by neurologists with extensive experience in MG diagnosis and management.”

Added text: line 145-148

“The evaluation of these clinical scores, as well as the determination of whether a patient had reached MM or better status (MM, Complete Stable Remission, or Pharmacologic Remission), was conducted by neurologists with expertise in MG management.”

Comment #3

What were the baseline characteristics of patients in the 2015 validation cohort? A table summarizing them, perhaps in the supplement, will enhance the findings in the study.

Response:

We thank the reviewer for this valuable and insightful suggestion. We have added a comprehensive supplementary table (Supplementary Table S1) summarizing the demographic and clinical characteristics of the validation dataset.

Added text: line 293-296

"The baseline characteristics of the validation cohort are presented in Supplementary Table S1. The validation cohort demonstrated similar demographic and clinical patterns to the training dataset, with comparable distributions of MG subtypes and clinical severity scores."

Reviewer #3:

Comment #1

The authors should provide a thorough review of existing models that predict MM status in MG patients.

Response:

We thank the reviewer for this important suggestion. After conducting a comprehensive literature review, we found that, to our knowledge, no previous studies have developed machine learning models specifically for MM status prediction in MG patients. This represents a significant gap in the literature that our study addresses.

Comment #2

If other models for MM prediction exist, the authors need to clearly highlight the advantages of their approach in comparison.

Response:

We thank the reviewer for this excellent point. While no machine learning models exist specifically for MM prediction, existing clinical approaches rely on single clinical score cutoffs, which have inherent limitations. To demonstrate the superiority of our approach, we compared our ensemble model performance with individual score cutoff methods in the validation dataset. Our ensemble model achieved an AUC of 0.94, demonstrating clear superiority over rule-based methods using individual scores: MGADL alone (AUC = 0.92), MGC alone (AUC = 0.91), and MGQOL15 alone (AUC = 0.85). This comparison clearly demonstrates the advantage of our integrated machine learning approach over traditional single-score cutoff methods (Fig 6).

Comment #3

(Line 249): The legend for Figure 2 appears after the legend for Figure 3. Please correct the order.

Response:

We thank the reviewer for identifying this formatting error. We have carefully corrected the figure legend order throughout the manuscript to ensure proper sequential numbering and presentation.

Comment #4

(Line 250): Change "he H matrix" to "The H matrix".

Response:

We thank the reviewer for catching this typographical error. This has been corrected.

Comment #5

(Line 261): The authors should report both training and test results for all four models, including SVM, logistic regression, random forest, and Naive Bayes.

Response:

We thank the reviewer for this important suggestion. We completely agree that comprehensive reporting of both training and test results for all models enhances the transparency and completeness of our evaluation. We have added detailed ROC curves showing both training and test results for all four individual models (SVM, logistic regression, random forest, and Naive Bayes) in the supplementary figures (S1-S3 Figures).

Comment #6

(Line 261): Since a 5-fold cross-validation was used, the variability across the five folds should be reported.

Response:

We thank the reviewer for this excellent point about reporting cross-validation variability. We have comprehensively reported the variability across all five cross-validation folds by showing individual fold results in our ROC curve figures (Supplementary Figures 1-3). Given the manageable number of folds (n=5), we present all individual AUC values for each fold rather than just summary statistics, providing complete transparency about model performance variability.

Comment #7

(Line 274): Similarly, for the ensemble model, both training and test performance should be shown, along with the variability across the five cross-validation folds.

Response:

We thank the reviewer for this important suggestion. We have included comprehensive results showing both training and test performance for the ensemble model, with individual fold results displayed in the ROC curves to demonstrate the complete range of performance across all five cross-validation iterations (Fig 5).

Comment #8

(Line 277): Given that five iterations were conducted, was an ensemble model generated for each iteration? If so, how were the five ensemble models applied to the external validation dataset? Please clarify.

Response:

We thank the reviewer for requesting this important methodological clarification. For external validation, we used all five trained models from each cross-validation fold: for each validation sample, we calculated predictions from all five folds and used soft voting (averaging probabilities) to generate the final ensemble prediction.

line 193-195:

“For performance evaluation including AUROC, we applied a soft voting strategy, averaging the predicted probabilities from all four models to generate the final prediction scores.”

Comment #9

Figures 4 and 5: The ROC curves shown in Figures 4 and 5 use inconsistent font sizes, legend locations, and titles. Please ensure uniform formatting.

Response:

We thank the reviewer for this attention to detail regarding figure presentation. We have carefully reviewed and uniformly corrected the formatting of all ROC curve figures, ensuring consistent font sizes, legend locations, and titles throughout the manuscript.

Attachment Submitted filename: Response to Reviewers.docx

# Peer review: Decision Letter 1

**DOI:** 10.1371/journal.pone.0330044.r003

**Authors:** Pinyi Lu

25 Jul 2025

Predicting achievement of clinical goals using machine learning in myasthenia gravis

PONE-D-24-57934R1

Dear Dr. Uzawa,

We’re pleased to inform you that your manuscript has been judged scientifically suitable for publication and will be formally accepted for publication once it meets all outstanding technical requirements.

Within one week, you’ll receive an e-mail detailing the required amendments. When these have been addressed, you’ll receive a formal acceptance letter and your manuscript will be scheduled for publication.

An invoice will be generated when your article is formally accepted. Please note, if your institution has a publishing partnership with PLOS and your article meets the relevant criteria, all or part of your publication costs will be covered. Please make sure your user information is up-to-date by logging into Editorial Manager at Editorial Manager®  and clicking the ‘Update My Information' link at the top of the page. For questions related to billing, please contact billing support .

If your institution or institutions have a press office, please notify them about your upcoming paper to help maximize its impact. If they’ll be preparing press materials, please inform our press team as soon as possible -- no later than 48 hours after receiving the formal acceptance. Your manuscript will remain under strict press embargo until 2 pm Eastern Time on the date of publication. For more information, please contact onepress@plos.org.

Kind regards,

Pinyi Lu, Ph.D.

Academic Editor

PLOS ONE

Reviewers' comments:

Reviewer's Responses to Questions

**Comments to the Author**

Reviewer #2: All comments have been addressed

Reviewer #3: All comments have been addressed

**********

2. Is the manuscript technically sound, and do the data support the conclusions?

Reviewer #2: Yes

Reviewer #3: Yes

**********

3. Has the statistical analysis been performed appropriately and rigorously?

Reviewer #2: Yes

Reviewer #3: Yes

**********

4. Have the authors made all data underlying the findings in their manuscript fully available?

The PLOS Data policy

Reviewer #2: Yes

Reviewer #3: Yes

**********

5. Is the manuscript presented in an intelligible fashion and written in standard English?

Reviewer #2: Yes

Reviewer #3: Yes

**********

Reviewer #2: The authors have adequately addressed my comments and revised the manuscript accordingly. The manuscript is acceptable for publication.

Reviewer #3: (No Response)

**********

what does this mean? ). If published, this will include your full peer review and any attached files.

If you choose “no”, your identity will remain anonymous but your review may still be made public.

**Do you want your identity to be public for this peer review?** For information about this choice, including consent withdrawal, please see our Privacy Policy

Reviewer #2: No

Reviewer #3: No

**********

# Editor report: Acceptance letter

**DOI:** 10.1371/journal.pone.0330044.r004

**Authors:** Pinyi Lu

PONE-D-24-57934R1

PLOS ONE

Dear Dr. Uzawa,

I'm pleased to inform you that your manuscript has been deemed suitable for publication in PLOS ONE. Congratulations! Your manuscript is now being handed over to our production team.

At this stage, our production department will prepare your paper for publication. This includes ensuring the following:

* All references, tables, and figures are properly cited

* All relevant supporting information is included in the manuscript submission,

* There are no issues that prevent the paper from being properly typeset

You will receive further instructions from the production team, including instructions on how to review your proof when it is ready. Please keep in mind that we are working through a large volume of accepted articles, so please give us a few days to review your paper and let you know the next and final steps.

Lastly, if your institution or institutions have a press office, please let them know about your upcoming paper now to help maximize its impact. If they'll be preparing press materials, please inform our press team within the next 48 hours. Your manuscript will remain under strict press embargo until 2 pm Eastern Time on the date of publication. For more information, please contact onepress@plos.org.

You will receive an invoice from PLOS for your publication fee after your manuscript has reached the completed accept phase. If you receive an email requesting payment before acceptance or for any other service, this may be a phishing scheme. Learn how to identify phishing emails and protect your accounts at https://explore.plos.org/phishing.

If we can help with anything else, please email us at customercare@plos.org.

Thank you for submitting your work to PLOS ONE and supporting open access.

Kind regards,

PLOS ONE Editorial Office Staff

on behalf of

Dr. Pinyi Lu

Academic Editor

PLOS ONE
